# Public communication and outreach by mosquito programs in the United States

**DOI:** 10.1371/journal.pgph.0003804

**Published:** 2024-12-19

**Authors:** Nicole Thomas, Jo Anne G. Balanay, Sachiyo Shearman, Stephanie L. Richards

**Affiliations:** 1 Department of Health Education and Promotion, East Carolina University, Greenville, North Carolina, United States of America; 2 School of Communication, East Carolina University, Greenville, North Carolina, United States of America; Arizona State University, UNITED STATES OF AMERICA

## Abstract

Blood feeding female mosquitoes cause itchy welts and can transmit pathogens that cause diseases such as chikungunya, malaria, West Nile encephalitis, and Zika. Mosquito control programs conduct mosquito, pathogen, and epidemiological surveillance, carry out source reduction, treat mosquito habitats with larvicides or adulticides, and disseminate information to the public. Here, 100 organizations (e.g., private/public mosquito control programs, national professional mosquito/pest control associations) in the United States were asked to complete a survey (N = 39 respondents) about their public communication and outreach efforts. Results indicate most programs (N = 27, 69%) have dedicated personnel for public communication. A checklist was constructed to compare communication strategies between a subset of program websites and Facebook pages. Recommendations for improving public communication and outreach strategies (e.g., digital tools, more frequent updates, public engagement strategies) for mosquito control programs are discussed.

## Introduction

Mosquitoes (Order Diptera; Family Culicidae) transmit pathogens that impact public and veterinary health (e.g., yellow fever [virus], dengue [virus], West Nile [virus], chikungunya [virus], filariasis [worm], and malaria [protozoan]) [1]. Mosquito control programs (MCP) protect public health from mosquito-related issues using integrated mosquito management methods such as community education, source reduction, bed nets, larvicides, adulticides, biological control, and release of sterilized or genetically altered mosquitoes [1–3]. Surveillance informs operational decisions of MCP and helps programs evaluate intervention efforts [4].

Social media is one of the largest growing forms of community outreach in the United States (US) and 93% of adults use the internet [5]. Facebook is the most used social media platform (68% of internet users) [6, 7]. In some cases, people prefer social media platforms over mainstream outlets as a news source due to a stronger sense of connection by direct association or perceived closeness [8, 9]. Program utilization of different media platforms including social media, podcasts, television, and websites could increase public knowledge and awareness of mosquito-borne diseases and methods for prevention. Internet-based sources include micro-blog posts (e.g., Facebook and X [formerly Twitter]), web encyclopedias, search queries, and other forms of social media [10]. These sources are especially helpful in areas where public health agencies are limited such as developing countries where traditional public health outreach methods may be lacking [10]. In some cases, public health agencies deal with public distrust of government officials to provide safe solutions (e.g., yellow fever vaccine) [9]. Media platforms are readily available to public health agencies; however, lack of real-time information distributed through these methods can lead to misinformation [11].

Through investigation and analysis of social media and other outreach methods used by county/state/federal agencies, private pest control companies, and professional public health pest control associations, public education about mosquito-borne disease and mosquito control could be streamlined and improved. There has not yet been another comparative analysis of such entities. Here, a knowledge gap is filled by finding similarities in effective communication methods between different types of pest-related agencies that can be synthesized and utilized by multiple agencies in the future. Improvement of outreach efforts can facilitate public communication by MCP and combat misinformation. Analysis of keywords in public social media messages can help identify early indicators to integrate infodemiology (i.e., online information about human behavior) and/or infoveillance (i.e., surveillance of online information) into public communication campaigns [10, 12]. Exploration of public search and commenting queries on social media outlets can show areas of rising public concern related to potential outbreaks. Google search demands have been previously analyzed for a flea-related disease outbreak of plague [13] and similar methods could be implemented for mosquito-borne diseases. Google Trends (GT) can track internet search history for a specified range of time using term and topic searches [13]. Spatiotemporal search parameters allowed GT to monitor relationships between public internet searches and confirmed plague cases in the region of interest [13].

Investigation into methods used by the public when conducting illness queries can be a starting point for surveillance data. Some of the public may post on Facebook to obtain opinions, while others may use Google or YouTube to search for information [8]. Some individuals search government health agency websites for information, and others utilize social media for broadcasting on X about symptoms [8]. The public generally seeks information when health risk uncertainty rises and may engage in popular social platforms (i.e., Reddit) for posting questions soliciting public response [8]. Emphasis placed on illness symptom searches or posts is “syndromic surveillance” [14]. On platforms such as X, an individual can post symptoms and their geo-location data is recorded [14]. By highlighting key words and emojis, symptoms can be tracked, and outbreak trends confirmed to evaluate risk [14]. Countries, such as the US, United Kingdom, and China track influenza trends by comparing social media trends with confirmed outbreaks [14]. The same study showed that, where locations were tracked, relevant tweets were positively correlated with observed public health data. On a larger scale, predictions of epidemics could be made from social media data which could help track transmission of vector-borne, food-borne, and other illnesses [10].

One obstacle facing public health educational campaigns is determining the best method(s) for disseminating information including evaluation of: 1) information source, 2) messaging, 3) audience, and 4) method of information delivery (e.g., news, blog, social media) [6, 15]. Hence, the evaluation of outreach methods used by public and private health agencies would be beneficial [6]. To combat misinformation, public health agencies should effectively distribute accurate information to the public [15]. The internet presence of public health agencies should engage the public through resources such as social media [15]. Public and private MCP must also engage with the public via social and traditional types of media and advertising must be balanced with reliable information to maintain credibility [8].

Public interaction strategies should provide up-to-date information and enhance engagement with social media posts [15]. Knowledge of diverse disciplines (e.g., business, psychology, marketing) can enhance public health messaging [6]. Scientists should succinctly relay findings to be received by audiences of different backgrounds and cultures to increase the likelihood of reading and sharing [16]. Improved marketing tools can help structure messaging to increase positive perception of information [6]. Public health organizations often disseminate information via academic journals and academic conferences [6]. Higher value is placed on information received by people with whom the recipient has an established relationship or familiarity [9]. Therefore, increasing internet and social media presence is vital for public health organizations to help establish themselves as consistent and knowledgeable sources of information to the community [15].

Daily news reporting is related to public internet searches; hence, these sources can be paired to streamline and improve communication [17]. This relationship was observed during the 2016 Zika virus outbreak and showed an avenue of information sharing to further mosquito control education [17]. The same study showed a link between news coverage of public health announcements and an opportunity to share information with the public about vector control during the initial outbreak period. Increasing public awareness of public health pests and mosquito-borne diseases can be a joint effort between different cooperating agencies and industries. Risk communication, messaging, and other forms of outreach can be improved when multiple specialties provide input that considers audience diversity. Through utilization of market research and business strategies, public health agencies can incorporate cost-effective communication methods to disseminate information [6, 16]. Social media outlets provide an opportunity for cost-conscious public health messaging [11]. Risk communication plans should be in place and implemented before an outbreak. Proactive evaluation of current methods of communicating public health pest information would inform policy recommendations to improve accessibility. This could involve setting standards for timely delivery of information and/or improving access to communication technologies in underserved communities. Consequently, the objectives of this study were to: 1) Identify and describe current public communication and outreach methods for disseminating information on mosquito-borne disease awareness and prevention by county/state/federal agencies, private vector/pest control companies, and professional public health pest control associations, and 2) Analyze communication and outreach methods regarding mosquito-borne disease to provide recommendations on improving future communication.

## Materials and methods

### Ethics statement

The survey was approved by the East Carolina University Medical Center Institutional Review Board (UMCIRB# 24–000301). Participants provided written consent prior to completing the survey.

### Survey on public communication

Mosquito control programs vary in size and scope across the US; hence, differences in public communication methods were expected between programs. To assess and compare MCP communication efforts, a 23-question survey was developed in Qualtrics and administered by email to 100 MCP and other mosquito-related organizations across the US from March 7–22, 2024 (S1 Appendix). The invitation was initially sent by email on March 7, 2024 and a follow-up reminder was sent on March 14, 2024. Survey recipients were primarily selected from the membership list of programs of the American Mosquito Control Association (AMCA) to ensure representation from different US regions (www.mosquito.org). Investigators also compiled and included contact information for private mosquito control agencies via website searches since these types of agencies are generally underrepresented in AMCA membership. Questions included topics such as geographic location, funding level, mosquito-borne diseases of concern, frequency/ types of communication utilized, status of communication staff, and availability of funding for communication.

The following types of MCP were contacted: state agency (SA), federal agency (FA), private pest control agency (PR), public pest control (PU), and professional public health pest control associations (PA) (Table 1). These types of programs are representative of the MCP landscape across the US. Programs in the following US regions (based on AMCA regions) were contacted: 1) North-Atlantic, 2) Mid-Atlantic, 3) South-Atlantic, 4) North-Central, 5) West-Central, 6) South Central, 7) North-Pacific, 8) South-Pacific/Pacific (https://www.mosquito.org/amca-regions/). One federal agency (no headquarter location provided) was also included (Table 1).

**Table 1:** Regions and agency types for 100 programs contacted within the United States.

Region	Total	States	State Agency (SA)	Federal Agency (FA)	Private Pest Control (PR)	Public Pest Control (PU)	Professional Public Health Pest Control Agency (PA)
North-Atlantic	10	Connecticut, Maine, Massachusetts, New Hampshire, New Jersey, New York, Pennsylvania, Rhode Island, Vermont	2	2	3	2	1
Mid-Atlantic	21	Delaware, District of Columbia, Maryland, North Carolina, Virginia, West Virginia	1	-	12	6	2
South Atlantic	10	Alabama, Florida, Georgia, South Carolina, Puerto Rico, US Virgin Islands	-	1	3	5	1
North Central	15	Illinois, Indiana, Iowa, Kentucky, Michigan, Minnesota, Missouri, Ohio, Tennessee, Wisconsin	-	1	1	12	1
West Central	10	Colorado, Kansas, Nebraska, New Mexico, North Dakota, South Dakota, Utah, Wyoming	-	-	-	9	1
South Central	13	Arkansas, Louisiana, Mississippi, Oklahoma, Texas	-	1	6	6	-
North Pacific	10	Alaska, Idaho, Montana, Oregon, Washington	1	-	1	8	-
South Pacific	10	Arizona, California, Hawaii, Nevada, American Samoa, Guam	3	1	2	3	1

### Website and social media analysis

A media content checklist was developed to evaluate a subset of respondent organizations for items such as: Feedback provided to the public, mosquito-borne disease awareness and prevention information, and messaging delivery systems/methods. This checklist allowed for the comparison of websites with social media resources and was developed based on expert opinion and a review of resources. Each organization’s posts were evaluated and investigators documented whether or not images, videos, positive framework, or negative framework messages were used in posts. It was documented whether or not organizations allowed public comments. To determine if feedback was provided to the public, all public comments were reviewed (where available) to determine if there was a reply from the MCP. The checklist assessed: 1) program purpose, 2) mosquito-borne disease (e.g., diseases of concern, numbers of cases/illness/death), 3) prevention strategies, and 4) information delivery methods (e.g., positive/negative messaging [benefits vs. risks], presence/absence of images, ability of audience to provide feedback). The checklist was completed for a subset of five websites and related Facebook pages including private pest control programs (this group was underrepresented in the survey respondents), municipal mosquito control, and statewide non-profit mosquito/pest association. These five organizations were randomly selected based on the presence of a website and Facebook page. Facebook pages were evaluated for posts made between January 1—December 31, 2023 (date range selected due to ease of monitoring and inclusion of all four seasons during a one-year period).

### Data analysis

A total of 39 (39% response rate) complete surveys were received in Qualtrics and data were analyzed with Statistical Package for the Social Sciences (SPSS 29, IBM, Armonk, New York). Chi-square (*P*<0.05) was used to assess the association between reported funding and personnel dedicated to risk communication.

## Results

Anonymized raw survey data are included as a supplementary file (S1 Data). Some respondents skipped questions; therefore, total numbers may vary in figures. No survey responses were received from private pest control programs. Respondents were from the following US regions and states: North-Atlantic (N = 2, 5%; New York), Mid-Atlantic (N = 3, 8%; North Carolina, Virginia), South-Atlantic (N = 5, 13%; Georgia, South Carolina), North-Central (N = 13, 33%; Illinois, Michigan, Missouri, Ohio, Wisconsin, Native American reservation between Wisconsin and Michigan), West-Central (N = 7, 18%; Colorado, Utah, Wyoming), South Central (N = 2, 5%; Louisiana), North-Pacific (N = 1, 3%; Washington), and South-Pacific (N = 6, 15%; Arizona, California).

**Fig 1:**
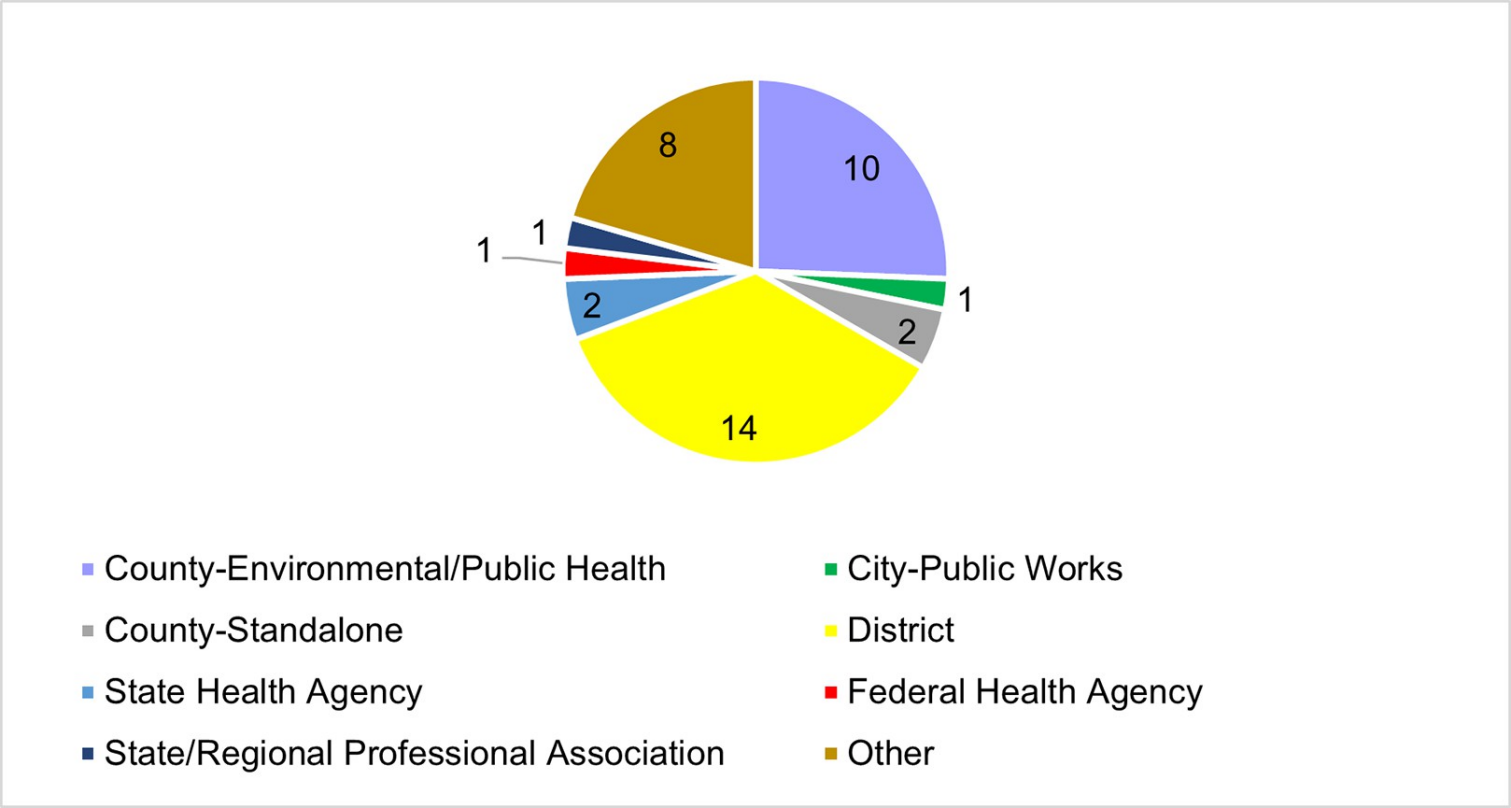
Program types of survey respondents.

Respondent programs included: district or county environmental/public health, other (e.g., Indian Health Service, US Environmental Protection Agency, and county mosquito programs through public works), state health, large county, state/regional professional association, federal health, and city public works (Fig 1). Programs reported an average of $900,000 in total annual program funding, ranging between $0 to $20,000,000. Some respondents (N = 20; 51%) indicated their program covered a larger region than where they were based, while others (N = 19; 49%) stated their program covered only the region in which they were located. Programs covering a larger region indicated coverage areas included other municipalities, counties, and multiple states.

Respondents (scale of 1–10; 1 = weak, 10 = strong) indicated an emphasis of 7.7 ± 2.4 (mean ± standard deviation) on information-sharing regarding the specific diseases of prevalence in their area. Examples of reasons for low ratings included, “we support other entities in doing this but do not do so ourselves”, “we have no one dedicated to public outreach and education”, and “we can do better”. No respondents select a value of 1. Low emphasis ratings (i.e., 2–4) comprised 18% of the total responses. Medium emphasis ratings (i.e., 5–7) were 8% of responses. Most (74%) indicated a high emphasis (i.e., 8–10) on information sharing.

Most respondents (N = 27; 69%) indicated having a dedicated public communication outreach division for dissemination of information about vector-borne disease and/or mosquito control. Five (15%) respondents indicated not having a public outreach division and one (3%) was unsure. However, 34% (N = 11) of respondents indicated their agency had funding dedicated to public communication, 53% (N = 17) did not have funding, and 13% (N = 4) were unsure. Dedicated communication funding was significantly associated with dedicated communication personnel (*Χ*^2^ = 43.04; *P*<0.01).

**Fig 2:**
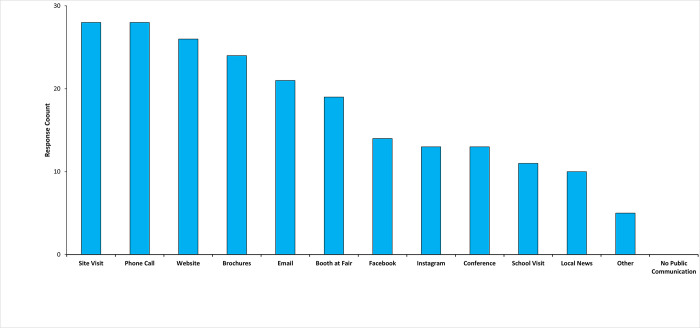
Methods of public communication used by respondents.

The primary communication methods indicated by respondents included response to citizen pest complaints via site visits (N = 28, 13%), phone calls (N = 28, 13%), and website (N = 26, 12%) (Fig 2). Social media outlets were used as follows: Facebook (N = 14, 7%) and Instagram (N = 13, 6%) (Fig 2).

**Fig 3:**
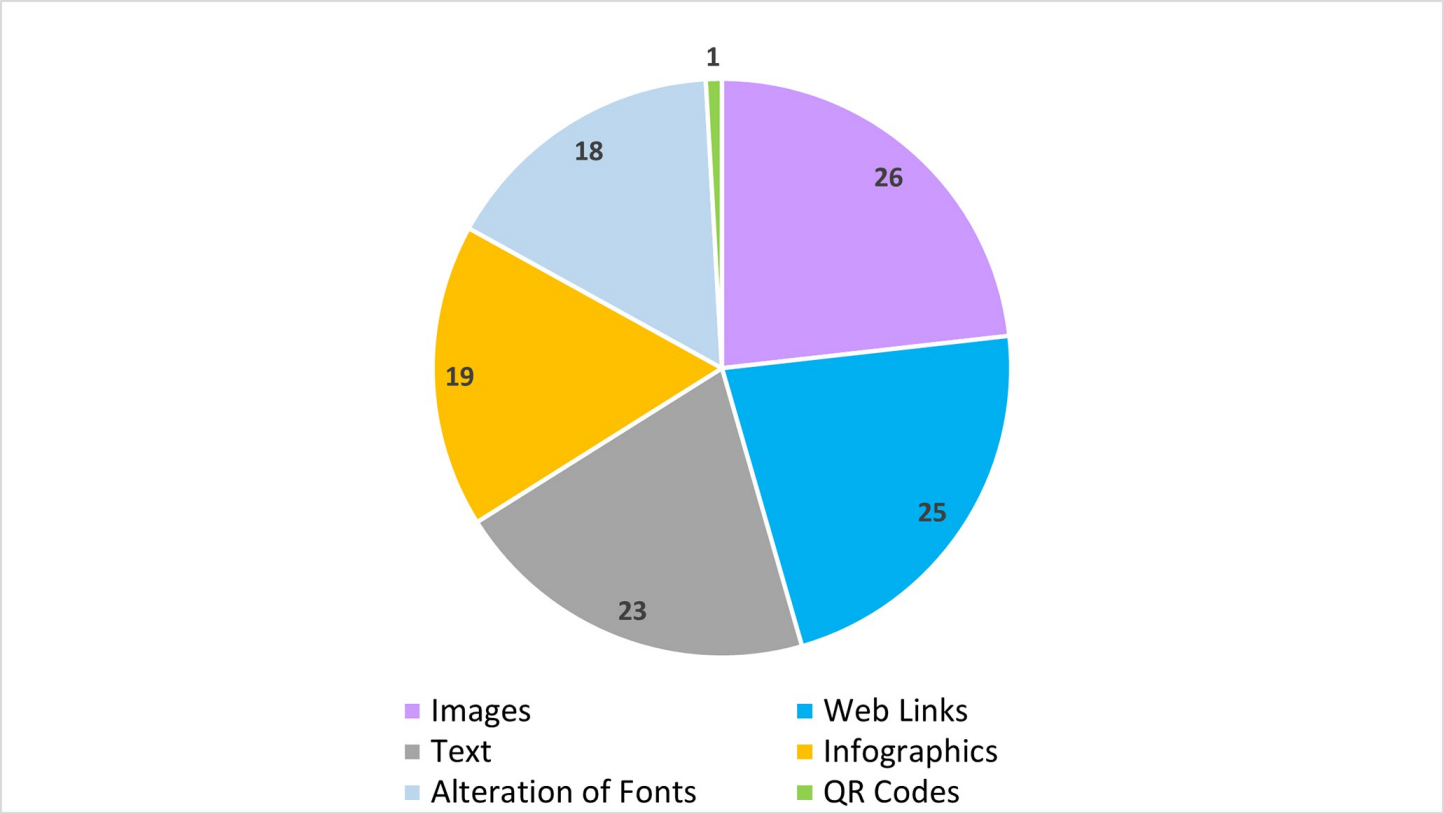
Frequency and types of communication used by mosquito control programs.

Fig 3 shows the frequency and types of communication used by MCP. Images were frequently used in online public communication messages, followed by web links, text, infographics, and alteration of fonts. One respondent reported using quick response (QR) codes. Respondents indicated that positive framework/phrasing was their primary delivery method (N = 19, 90%) while negative framework (N = 6, 29%) was used less frequently. In some cases, audiences could provide feedback (N = 8, 38%) and paid advertisements (N = 4,19%) were also utilized. When customizing information for social media, programs shorten messaging (N = 14, 50%), modify messages for the target audience (N = 11, 39%), or make no modifications to messaging (N = 10, 36%). Some respondents indicated restricting public comments on social media (N = 4, 14%) and/or selected ‘other’ (i.e., messaging handled by a public information officer or is posted on their website [N = 2, 7%]).

**Fig 4:**
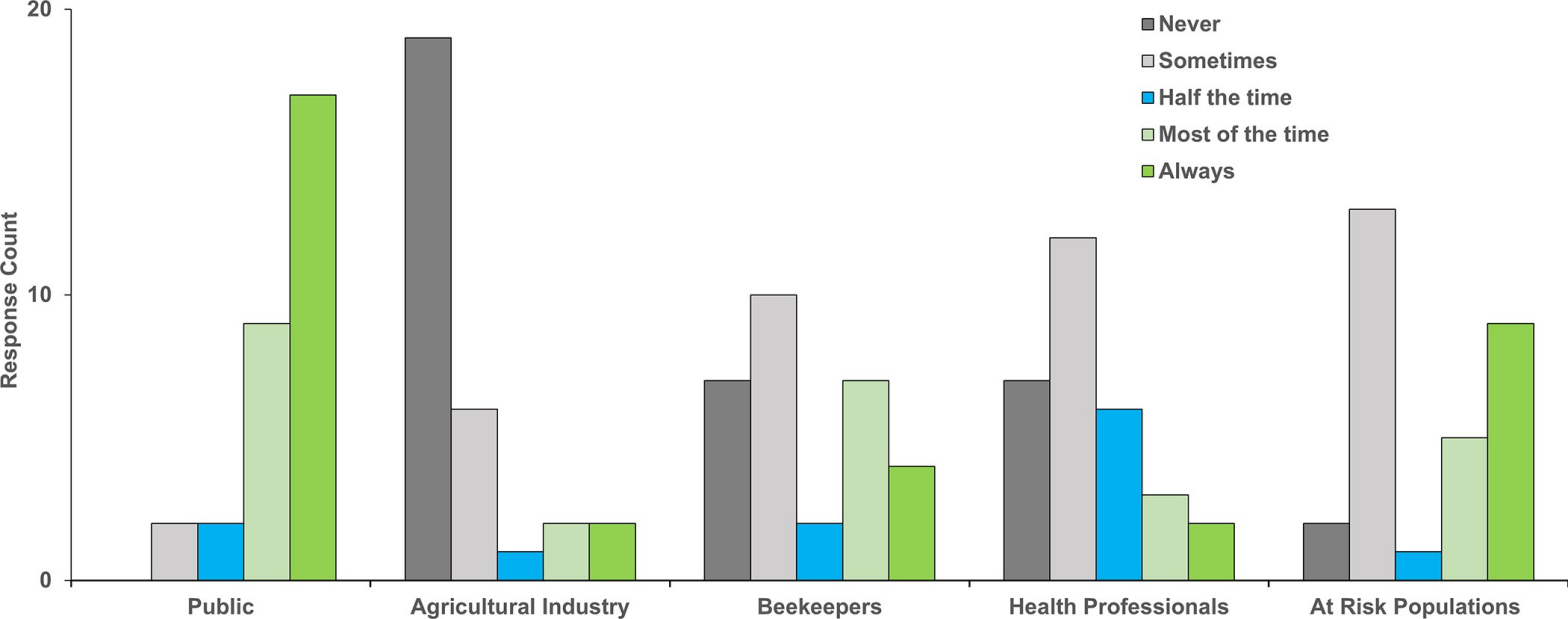
Frequency of mosquito control program communication with different groups.

Fig 4 shows the frequency of MCP communication with different groups including the public, agricultural industries, beekeepers, health professionals, and at risk populations (e.g., elderly, immunocompromised). Most respondents indicated communicating with the public always, but never with agricultural industries. Many MCP communicated with beekeepers, health professionals, and at risk populations sometimes. Programs not communicating with the public listed these barriers: lack of time (N = 11, 25%), lack of communication personnel (N = 11, 25%), lack of funding (N = 8, 18%), lack of social media expertise (N = 8, 18%), and/or perceived lack of public interest (N = 2, 5%).

A checklist was completed to analyze program websites and Facebook pages for five pest control programs over a one-year period. An additional evaluation was carried out on the most active Facebook post. Media content analysis included private pest control companies (N = 3, 60%), city mosquito control (N = 1, 20%), and a statewide non-profit mosquito/pest association (N = 1, 20%). Programs showed information about mosquito borne diseases (N = 3, 60%) where diseases of concern (N = 2, 40%) and total number of illness/deaths (N = 1, 20%) were listed. Most (N = 3, 60%) did not include prevention strategies to protect against mosquito-borne illness. Most Facebook pages included prevention strategies (N = 4, 80%) that focused on treatment options and sales and allowed the public to leave comments. One organization blocked the ability for the public to respond/comment. All programs provided images on their website and Facebook page, including mosquitoes/vectors (N = 4, 80%), treatments (N = 3, 60%), positive people (N = 2, 40%), and the environment (N = 1, 20%).

The Facebook analysis showed that all programs (N = 5, 100%) provided information in a positive framework whereas one program (20%) also used negative framework in their messaging. All websites (N = 5, 100%) showed a statement of purpose and links to additional information on external websites. However, 60% (N = 3) of Facebook pages did not include a statement of purpose. The average number of agency posts on all five Facebook pages during the study period was (mean ± standard deviation) 58.6 ± 47.1 and 51.7 ± 85.3 images were shared via Facebook posts. When the most popular post (21 likes/comments/shares ± 24.5) on each program’s Facebook page was analyzed, 57% (N = 4) of the posts focused on public participation (e.g., community meeting), while others included information about disease prevention and awareness.

## Discussion

Mosquito control programs communicate with the public in a variety of ways including, but not limited to television, radio, newspaper, website, and social media [18]. Social media showed an increase in communication frequency between the public and health organizations during the Zika epidemic in 2015–2016 [19]. Social media allows individuals not connected to traditional media sources to remain informed [3]. Web links can be provided for users to find additional information and this method is frequently utilized [3]. Environmental health literacy can facilitate risk communication by helping vector control programs address audiences from a variety of backgrounds [20]. This study showed that site visits and phone calls initiated by the public were the most utilized methods of public communication, and that social media outlets were underutilized for reaching a larger audience. Potential barriers that might have contributed to lack of social media usage include budget constraints, audience demographics, and organizational capacity. Organizations involved in information dissemination on mosquito control and mosquito-borne disease prevention should give more attention to improving social media use as a potentially efficient and effective communication tool for the public.

Previous studies show low survey participation in states having MCP with lower budgets, which may have also limited survey responses here [21]. Limited funding may have also contributed to the lack of survey participation by private pest control companies, where funding efforts are likely weighted towards field personnel and equipment [21]. This should be investigated further. Our results indicate funding for public communication was related to the presence of dedicated communication personnel, which may imply the importance of financial resources in improving an organization’s communication and outreach program. It was encouraging that most indicated having dedicated public communication outreach division; however, more than half did not have funding specifically tagged to support this. A variety of communication methods are used by programs with the most frequent being site visits and/or phone calls responding to citizen complaints about mosquito issues. This type of interaction shows the public is aware of MCP and the services they provide and provides an opportunity for programs to share knowledge about mosquito control and related diseases. A study on public perception of mosquito control in Florida found most respondents never or rarely searched for information on mosquito control or related disease topics; hence, designing communication efforts (e.g., neighborhood-level efforts, public information sessions, social media) to reach passive audiences and convey the seriousness of the topic is important [22]. The same study indicated that outreach should also focus on how residents can help control mosquitoes (e.g., source reduction) and this was especially useful for homeowners. Another study showed increased communication to educate the public on mosquito biology, how to prevent mosquito bites, and aspects of mosquito control methodology could impact public perception of risk for factors such as exposure to mosquito-borne disease and pesticides [23]. Our finding that most websites and/or social media sites focused on mosquito control treatment rather than mosquito-borne illness prevention indicates an area that could use improvement so that both aspects of public health protection are promoted. There is generally public support for mosquito surveillance, but not pesticide use [23]. When the public had limited or no knowledge of public or veterinary health risks from mosquito-borne diseases, they were against pesticide use [23]. The same study showed public support for pesticide use increased for groups that understood the health risks of mosquito-borne disease. It is important that MCP know how to use social media to share mosquito-borne disease and other information in a timely and trustworthy manner [18]. Establishing trust with the public audience regarding sharing mosquito control information whether in person or online should be accomplished in advance of an emergency (e.g., outbreak, hurricane) situation [24].

### Study limitations

Although we had a relatively high (39%) survey response rate from a variety of different types of programs across the US, it was difficult to recruit private pest control companies to complete the survey due to lack of direct contact information. Other surveys carried out with NC (30% response) or US (26% response) MCP showed similar response rates [21, 25]. Program funding reported by respondents varied widely between programs (range, 0 to $20 million) and budgetary constraints for communication efforts varied, likely due to program size and priorities. Analysis of a subset of programs’ social media and websites was successful but may be limited due to the willingness of the public to engage with posts [11]. Even if someone positively views a post, they may or may not respond to the post [11]. This study did not consider the number of times a post was viewed but this may be considered if other social media platforms are analyzed.

### Future studies

Information about public communication methods of private pest control programs should be studied further and compared to public programs. Future studies may consider implementing public communication strategies and assessment of efficacy within different types of audiences [20]. It would be beneficial for future research to expand the number of organizations whose media content is analyzed through checklists, including county, state, and federal programs.

## Conclusions and recommendations

Our results show communication personnel existed in most surveyed MCP and many respondents use social media as a communication tool. Several organizations prioritize communication about mosquito control and mosquito-borne diseases; however, these efforts are often handled by a public information officer or at a federal rather than regional level. Notably, this study found that the primary reasons for lack of online communication are due to the lack of personnel with social media knowledge and lack of personnel dedicated to communication, hence this deficiency could be improved with employee training on public communication via social media or other digital tools. Mosquito control programs could consider redistributing funding and/or priorities to increase public risk communication efforts. Posting more frequent updates about disease prevention and community involvement in advance of emergencies would likely help programs increase public engagement, awareness, and possibly long-term support for programs.

## Supporting information

S1 Data Anonymized raw survey data from respondents from United States mosquito control programs. (XLSX)

S1 Appendix Survey questions. (DOCX)
