# Supplementary material for: Public communication and outreach by mosquito programs in the United States
Source: PLOS Glob Public Health. 2024 Dec 19;4(12):e0003804. doi: 10.1371/journal.pgph.0003804 (PMC11658484; doi:10.1371/journal.pgph.0003804)
Supplement: S1 Appendix — (DOCX) [file pgph.0003804.s002.docx]

# **Appendix**

## Survey Questions

1. Please list the location information for your mosquito control or other pest/vector-related program:

City: ___________________________

County: _________________________

State: __________________________

1. Does your program cover a larger region or area (beyond the area of program residence)? If yes, please describe.
   - Yes

If yes, please describe: ________________________________

- - No

1. Please select the choice which best describes your program type:

- County mosquito control program within environmental/public health department
- City mosquito control program within public works department.
- Large county mosquito control effort
- Mosquito control district
- State health agency
- Federal health agency
- State or regionally funded professional association
- National professional association
- Non-profit mosquito/ pest association
- Private pest control company
- Other: _____________________. Please describe.

1. On the scale of 1 to 10, 1 = low emphasis and 10 = strong emphasis, how much does your program emphasize public communication regarding specific mosquito-borne disease(s) and/or other vector-borne diseases in your region?
2. Please explain why you selected your emphasis rating __________________

______________________________________________________________

1. What is the approximate total annual budget of your vector control program?

______________________________________________________________

1. Please list the approximate percentage of vector control program budget dedicated to each of the following:
   - Public education
   - Mosquito or other pest surveillance
   - Arbovirus surveillance: List arboviruses surveyed:

________________________________________________________

- - Mosquito control (e.g., larvicide, adulticide, source reduction)
  - Control of other vectors/pests (Please specify.)
  - Other: ___________________________________________________

1. From what source does your program receive funding?

If funding comes from a range of sources, please list approximate percentage received from each source below.

- Donations: _____________________________________________
- City/County Government: __________________________________
- State Government: _______________________________________
- Federal Government: _____________________________________
- Federal, State, or Local Government Grant (Please Specify): ______ _______________________________________________________
- Other: __________________________________________________
- Unsure/Don’t know

1. Does your organization or program have a division, group, and/or individual personnel dedicated or partially dedicated to public communication/outreach?

- Yes

If yes, please specify: _________________________________________

- No
- Unsure/ Don’t Know

1. Does your program have dedicated funding for public communication efforts about vector-related issues (e.g. mosquito-borne or other vector-borne diseases, mosquito control treatments, etc.)?

- Yes
  - If yes, approximately how much funding is dedicated to communication? Please describe.
  - No
  - Unsure/ Don’t Know

1. What are the primary methods of public communication utilized by your program? Please assign the items below a number, based on your program’s frequency of use, where 1 is the most frequently utilized. Please list 0 for items that do not apply.:

- Facebook
- Instagram
- Email
- Website
- Response to citizen pest complaints via site visit and/or in person consultation
- Response to citizen pest complaints via phone
- Workshop/ Conference
- Booth at local fair
- Visit to schools for education
- Brochures
- Local media outlet advertisements (e.g., regional news broadcasts, local newspapers, etc.). Please list and describe.
- My program does not conduct public communication.
- Other: _____________________. Please describe.

1. If your program conducts public communication, what types of messaging are used? Please assign the items below a number, based on your program’s frequency of use, where 1 is the most frequently utilized. Please list 0 for items that do not apply. Beside each applicable category, please specify if this communication is virtual or in-person:

- Informational (related to a notable subject or specific pest)
- Risk communication (notifying the public of an impending health risk/concern facing members of the community)
- My program does not conduct public communication.
- Other: _____________________. Please describe.

1. If your program conducts online public communication, approximately how frequently does your program update communication messaging? Select one and please specify if this communication is virtual or in-person:

- Once or more per week.
- Biweekly
- Monthly
- Seasonally
- Bi-annually
- Annually
- As needed if there is a disease outbreak
- As needed to provide details about mosquito control treatments
- My program does not conduct online public communication.
- Other: _____________________. Please describe.

1. Please describe program plans (if any) to initiate or further develop mosquito/pest communication. _____________________________________________________________
2. If your program does not conduct public communication, please describe barrier(s) that may impact this. Choose all that apply:

- Lack of time
- Lack of funding
- Lack of/limited personnel dedicated to communication.
- Lack of personnel expertise in social media or other online communication skills.
- Perceived lack of public interest.
- Other: _____________________. Please describe.

1. What communication platforms are utilized by your program?
   - In-person/Phone Outreach
   - Printed Brochures/Advertisement
   - Virtual Advertisement
   - Program Website
   - Social Media
   - Other – Please specify: ________________________
2. What is the approximate percentage of communication that is Virtual or Online versus In-Person/Phone Calls?
   - Virtual or Online: _____________________________________________
   - In-Person/ Phone: ____________________________________________
3. How is communication modified across media platforms used by your program? Select all that apply:
   - Abbreviated message for social media
   - Comments restricted on social media
   - Messages modified to reflect target audience
   - Other – please explain. ___________________________
   - No modifications
4. What items are included in your public outreach messages? Select all that apply.
   - Test
   - Infographics
   - Images
   - Font alteration (e.g. change of colors, increasing boldness, including italics)
   - Using web links
   - Other – please specify. __________________________________
5. Please describe your communication methods regarding delivery. Select all that apply.
   - Positive Framework/Phrasing
   - Negative Framework/Phrasing
   - Paid advertisements. Please specify (e.g. billboards, vehicle decals, virtual ads) __________________________________
   - Allowing audience feedback – please specify if this is via survey, phone calls, or public comments
6. How often do you target the following audiences with your mosquito/vector-related information?

- General public
- Never, Rarely, Sometimes, Often, Always
- Health professionals
- Never, Rarely, Sometimes, Often, Always
- At risk populations (e.g., immunocompromised, elderly people sensitive to insecticides)
- Never, Rarely, Sometimes, Often, Always
- Beekeepers
- Never, Rarely, Sometimes, Often, Always
- Agricultural Industries
- Never, Rarely, Sometimes, Often, Always
- Other (Please describe.): ______________________________

1. Please provide any additional feedback on communication here: ________________________________________________________
